# Conjugal Transfer of the Pathogenicity Island ROD21 in *Salmonella enterica* serovar Enteritidis Depends on Environmental Conditions

**DOI:** 10.1371/journal.pone.0090626

**Published:** 2014-04-04

**Authors:** Francisco J. Salazar-Echegarai, Hugo E. Tobar, Pamela A. Nieto, Claudia A. Riedel, Susan M. Bueno

**Affiliations:** 1 Millennium Institute on Immunology and Immunotherapy, Departamento de Genética Molecular y Microbiología, Facultad de Ciencias Biológicas, Pontificia Universidad Católica de Chile, Santiago, Chile; 2 Millennium Institute on Immunology and Immunotherapy, Departamento de Ciencias Biológicas, Facultad de Ciencias Biológicas y Facultad de Medicina, Universidad Andrés Bello, Santiago, Chile; 3 INSERM UMR 1064, Nantes, France; University of Helsinki, Finland

## Abstract

Unstable pathogenicity islands are chromosomal elements that can be transferred from one bacterium to another. *Salmonella enterica* serovar Enteritidis (*S.* Enteritidis) is a pathogenic bacterium containing such unstable pathogenicity islands. One of them, denominated ROD21, is 26.5 kb in size and capable of excising from the chromosome in certain culture conditions, as well as during bacterial infection of phagocytic cells. In this study we have evaluated whether ROD21 can be effectively transferred from one bacterium to another. We generated a donor and several recipient strains of *S.* Enteritidis to carry out transfer assays in liquid LB medium. These assays showed that ROD21 is effectively transferred from donor to recipient strains of *S.* Enteritidis and *S.* Typhimurium. When *Escherichia coli* was used as the recipient strain, ROD21 transfer failed to be observed. Subsequently, we showed that a conjugative process was required for the transfer of the island and that changes in temperature and pH increased the transfer frequency between *Salmonella* strains. Our data indicate that ROD21 is an unstable pathogenicity island that can be transferred by conjugation in a species-specific manner between *Salmonellae*. Further, ROD21 transfer frequency increases in response to environmental changes, such as pH and temperature.

## Introduction

Bacterial genomes evolve by various processes, such as mutations, chromosomal rearrangements, deletions of one or more genes or acquisition of foreign genetic material by horizontal gene transfer (HGT) [1]. The loss or gain of genetic material generates genomic changes that can rapidly and radically alter the lifestyle of the bacterium [2], [3]. These appear to be key mechanisms used by bacteria to genetically adapt to new environments, which can have a strong impact for the emergence of new pathogenic versions of a particular bacterial strain. The processes of horizontal gene flow include mobile genetic elements, such as conjugative plasmids, transposons, insertion sequences, prophages, integrons and genomic islands. *Salmonella enterica* is one of the most interesting examples of HGT, due to the large number of plasmids, genomic islands and prophages that these pathogenic bacteria contain [4]. *Salmonella enterica* is a facultative intracellular enteric pathogen that affects a wide host range and has more than 2,500 serovars, of which the serovars Typhimurium and Enteritidis are of great impact in health in humans, as well as in animals of commercial importance.

The availability of the complete genome sequence of several *Salmonella enterica* serovars has allowed comparative genomic analyses that led to the identification of regions acquired through HGT. One of such HGT-acquired regions is ROD21 (Region of Difference 21, [5]), which is a 26.5 kb genomic island, located between the 2,061,170 and 2,087,657 coordinates of the *S.* Enteritidis genome (NCTC13349). ROD21 can also be found in the genomes of *S.* Gallinarum, *S.* Dublin and *S.* Typhi [5]. The ROD21 region shows features that are characteristic of a pathogenicity island, such as insertion near to three copies of the gene coding for asparagine tRNA (*asnT*); a gene coding for an integrase at the 5′ end, similar to those present in phage P4; the region is flanked by direct repeat sequences (DRS) of 24 bp, which are identical to the last 22 bp of the gene coding for *asnT*; and a G+C percentage equal to 37.5% that is significantly different to the rest of the *Salmonella* genome (52.2%) [5], [6]. Further, ROD21 is considered a pathogenicity island because it contains the gene *tlpA*, which encodes a protein involved in the virulence of the bacterium through modulation of the host defense mechanisms that determine the regulation of NF-κB and caspase-1 activation [7]. Furthermore, recent studies have suggested that other genes encoded in this pathogenicity island are required for *S.* Enteritidis to cause disease in mice [6], [8]. Previous work from our laboratory have shown that ROD21 is an unstable pathogenicity island that excises from the chromosome by site-specific recombination, a process catalyzed by the P4 integrase and a putative excisionase (SEN1998), also encoded in this region [6], [9]. These enzymes are thought to recognize DRSs that flank the pathogenicity island [10] and produce a circular intermediate, which can either remain as an episomal element inside the bacterium or be degraded and disappear. Despite of this fact, previous studies have described that most clinical strains of *S.* Enteritidis isolated in various countries carry this pathogenicity island [6], [11], suggesting that it is possible that ROD21 can be transferred between *S.* Enteritidis strains. Consistent with this notion, bioinformatic analyses have identified ORFs in this pathogenicity island that show sequence identity with proteins involved in conjugative mobilization of DNA, such as genes found in several conjugative plasmids, such as MobA and TraD [6].

Based on the evidence described above, here we have determined whether this unstable pathogenicity island is transferable to recipient bacteria *in vitro*. Further, we have measured the transfer frequency and identified the mechanism responsible for this process. We observed that ROD21 could be transferred by conjugation between *S.* Enteritidis and between *S.* Enteritidis and *S.* Typhimurium. In addition, the frequency of ROD21 transfer can be modulated by changes in pH and temperature. Our data suggest that ROD21 can be transferred by conjugation between different *Salmonella* strains in a species-specific manner, a process that could be modulated by the environmental conditions confronted by bacteria.

## Materials and Methods

### Ethics Statement

All experimental procedures performed in this study were revised and approved by the Bioethics and Biosafety Committee of the School of Biological Sciences, Pontificia Universidad Católica de Chile. All animal work was performed according to the Guide for Care and Use of Laboratory Animals (National Institute of Health, USA) and Institutional guidelines. All experiments using animals were overseen by a Veterinarian at all times.

### Strains and plasmids

Strains used in this study are listed in Table 1. *Salmonella enterica* serovar Enteritidis PT1 (*S.* Enteritidis) was provided by Mrs. Alda Fernández from Public Health Institute of Chile, Santiago, Chile; *S.* Enteritidis PT4 was provided by Dr. Carlos Santiviago from University of Chile, Santiago, Chile; *Salmonella enterica* serovar Typhimurium (*S.* Typhimurium) 14028s was provided by Dr. Guido Mora from University Andrés Bello, Santiago, Chile; and *S.* Typhimurium pSLT^−^, was provided by Dr. Josep Casadesús from Universidad de Sevilla, Sevilla, Spain. *E. coli* strains H10407 and *E. coli* EDL933, were both provided by Dr. Roberto Vidal from University of Chile and *E. coli* strain DH5α (nalidixic acid resistant bacteria, NalR) was purchased from Invitrogen. These strains were stored at −80°C in LB medium supplemented with 20% glycerol and grown in liquid LB medium at 37°C with aeration provided by a shaker. Plasmids pKD3, pKD4 and pKD46 were obtained from the *E. coli* Genetic Resources at Yale CGSC, The Coli Genetic Stock Center. Plasmid pCLF1 was provided by Dr. Carlos Santiviago from the University of Chile, Santiago, Chile.

**Table 1:** Strains and plasmids used in this study.

Strains	Relevant genotype	Reference
***Salmonella enterica***		
*S.* Enteritidis PT4	Reference strain NCTC 13349	[5]
*S.* Enteritidis PT1	Clinical isolate, wild type strain	[6]
*S.* Enteritidis PT1 ROD21::*aph*	*S.* Enteritidis strain with *aph* gene inserted between 1975–1976 ORFs	This work
*S.* Enteritidis PT1 ROD2 pBAD-SEN1998	*S.* Enteritidis strain that carries pBAD-SEN1998 plasmid	[9]
*S.* Enteritidis PT1 ROD21::*aph* pBAD-SEN1998	*S.* Enteritidis strain with *aph* gene inserted between 1975–1976 ORFs and carries pBAD-SEN1998 plasmid	This work
*S.* Enteritidis PT1 ΔROD21 putAP::*cat*	*S.* Enteritidis strain lacking ROD21 and the *cat* gene inserted between *putA* and *putP* genes	This work
*S.* Typhimurium	Reference strain ATCC 14028s, wild type	[38]
*S.* Typhimurium putAP::*cat*	*S.* Typhimurium strain with the *cat* gene inserted between *putA* and *putP* genes	
This work		
*S.* Typhimurium pSLT^−^	*S.* Typhimurium strain that lacks the virulence plasmid	[16]
*S.* Typhimurium pSLT^−^ phoN::*dhfr*	*S.* Typhimurium strain that lacks the virulence plasmid and is resistant to trimethoprim	This work
***Escherichia coli***		
H10407	Clinical isolate enterotoxigenic pathotype, wild type strain	[39]
H10407 putAP::*cat*	*E. coli* strain with *cat* gene inserted between *putA* and *putP* genes	This work
EDL933	Clinical isolate enterohaemorragic pathotype, wild type strain	[40]
EDL933 putAP::*cat*	*E. coli* strain with *cat* gene inserted between *putA* and *putP* genes	This work
DH5α™	*E. coli* strain (F– Φ80*lac*ZΔM15 Δ(*lac*ZYA-*arg*F) U169 *rec*A1 *end*A1 *hsd*R17 (rK−, mK+) *pho*A *sup*E44 λ– *thi*-1 *gyr*A96 *rel*A1)	Invitrogen
**Plasmids**		
pKD3	Plasmid that contains *cat* gene	[12]
pKD4	Plasmid that contains *aph* gene	[12]
pKD46	Plasmid that contains Lambda red recombinase genes from phage Lambda	[12]
pCLF1	Plasmid that contains *dhfr* gene	GenBank: HM047090.1

Donor strain (*S.* Enteritidis ROD21::*aph*) and recipient strains of ROD21 (*S.* Enteritidis ΔROD21 *putAP::cat*, *S.* Typhimurium *putAP::cat*, *S.* Typhimurium pSLT^−^ *phoN::dhfr*, H10407 *putAP::cat*, EDL933 *putAP::cat*) were generated by Lambda Red-mediated recombination, as described by Datsenko and Wanner [12]. Briefly, a PCR product encoding *cat*, *aph* or *dhfr* gene was generated by PCR amplification from pKD3, pKD4 or pCLF1 plasmid, respectively. The primers used in this study are listed in Table 2. In order to insert the *aph* gene into ROD21, we used primer ROD21::kan (H1+P1) and primer ROD21::kan (H2+P2) to generate a PCR product that was inserted between genes SEN1975 and SEN1976. We also designed a set of primers to insert the *cat* gene into the chromosome of a *S.* Enteritidis PT1 strain that spontaneously lost ROD21, as described before [6]. These primers were putAP::Cm (H1+P1) and primer putAP::Cm (H2+P2) (Table 2). To insert the *cat* gene into *S.* Typhimurium 14028s chromosome we designed primers STM14028 putAP::Cm (H1+P1) and STM14028 putAP::Cm (H2+P2) (Table 2). To insert *dhfr* gene upstream to *phoN* gene into *S.* Typhimurium pSLT^−^ we designed primers STM_phoN_tmtp (H1+P1) and STM_phoN_tmtp (H2+P2) (Table 2). We also designed a set of primers to insert *cat* gene into *E. coli* H10407 and *E. coli* EDL933 strains. These were EHEC-ETEC putAP::Cm (H1+P1) and EHEC-ETEC putAP::Cm (H2+P2) (Table 2). The first 40 bp of all these primers aligned with the chromosome of the manipulated strains and the last 20 bp of these primers aligned with pKD3, pKD4 or pCLF1. Competent strains harboring the thermosensitive plasmid pKD46 were prepared as previously described [12] and PCR products containing *cat*, *aph* or *dhfr* genes were electrotransformed into these competent cells. After electrotransformation, bacteria were incubated for 1 h at 37°C with aeration in 1 mL of LB medium and then seeded in solid LB medium supplemented with 50 µg/mL kanamycin, 10 µg/mL chloramphenicol or 20 µg/mL trimethoprim. To verify the correct insertion of *aph* gene in the donor strain, a PCR amplification was performed with genomic DNA of the mutant strains and primers SEN1975 Fw and ROD21::kan (H2+P2). This PCR reaction generated a product of 2,388 bp only if the *aph* gene was inserted in the right position inside ROD21.

**Table 2:** Primers and probes used in this study.

Primers used to verify the genotype		
N°	Primer	Sequence (5′→3′)
1	SEN 1968 Fw	GACGGAAATCTTTTCGCCTG
	SEN 1970 (integrase) Rev	CGGCGTAATCTTCTGACCAT
2	SEN 1999 Fw	CAGCAAGACCCTGCCAGAGT
	SEN 2001 Rev	AGTGGGCTTATTGGGATCGG
3	SEN yeeO Fw	TCATAATCACCAGCGACTGG
	SEN 2013 Rev	CGCTCCAGCACCTCATTAAC
4	putAP Fw	ATGTGACCTGCGTTGCAAGC
	putAP Rev	GCCCCTTGAGCATGTCGACA
5	SEN1975 Fw	TTCTGATGAGCAGCGTAAAGAGGC
	SEN1976 Rev	TGGTGGTGGAAAGAATGATCACTT
6	LepA Gifsy-1 Fw	GCCCAGGTAGTTATCGAACCAGGA
	GogB Gifsy-1 Rev	GGTTTGATGTCGCCTCGTTACT

Primers used to insert resistance genes	
Primer	Sequence (5′→3′)
SEN 1970 (H1+P1)	ATGCTGGTAAAGCTGGTTCGATTTGTGTTTTACCAGCACGgtgtaggctggagctgcttc
asnT – SEN 1970 (H2+P2)	TTGTTTCTGAGCAAACTCAAACGGCCTGACCATCTCATACcatatgaatatcctcctta
putAP::Cm (H1+P1)	AAATTGTTAAAAAAAGAAAGAGGGAGTCTGACGGGGAAAAgtgtaggctggagctgcttc
putAP::Cm (H2+P2)	TCACAATCGATTTAACACACCATTTACATCAAATTTGATTtccatatgaatatcctcctt
SEN 1980::Kan (H1+P1)	AAAACTAACGGCAGCATTTATATTTGAGGTTAATTTATTAgtgtaggctggagctgcttc
SEN 1980::Kan (H2+P2)	CCAGTTATGGAGCTTAAATAAAAATGTATTTTCTATTTTGcatatgaatatcctccttag
EHEC-ETEC putAP::Cm (H2+P2)	ATTCACAATCGATTTAACACACCATTTACATTAAATTTTAccatatgaatatcctcctta
ROD21::kan (H1+P1)	GGAACTCTCCATTGGAGAGAATACATATCACTTGGGAAAAgtgtaggctggagctgcttc
ROD21::kan (H2+P2)	ATGTTTGTGTTTAAACATTATAATAAAATTTAACTTTTAAccatatgaatatcctcctta
STM 14028 putAP::Cm (H1+P1)	AAAAGGTGCAACCGCAAAAAATGTGAGAGAGTGCAACCTGaacttcatttaaatggcgcg
STM 14028 putAP::Cm (H2+P2)	CGTGGCAAGGGTGTTATGTTTACCAGGGCGACCGTATCCTggcgcgcctacctgtgacgg
STM_phoN_tmtp (H1+P1)	CAGACCGGTTTTACCGCTTCAGCCGGACAACGACGTCATGtcaagatcccctcacgctgc
STM_phoN_tmtp (H2+P2)	GCGGCCGGGCGTGGAGAATGCCTTTGGTTTCCCCGATTCGagagcgcttttgaagctggg

Primers and probes used to detect *attB1*	
attB1-nested-RT-Fw	GTTACTATGCGCCCCGTTCACAC
attB1-nested-RT-Rev	CCGATTAAGCCCCAAAAACTATG
rpoD-RT-Fw	GTTGACCCGGGAAGGCGAAA
rpoD-RT-Rev	CAGAACCGACGTGAGTTGCG
attB-1 probe	TTCGAGTCCAGTCAGAGGA
rpoD probe	CGACATCGCTAACG

**Figure 1:**
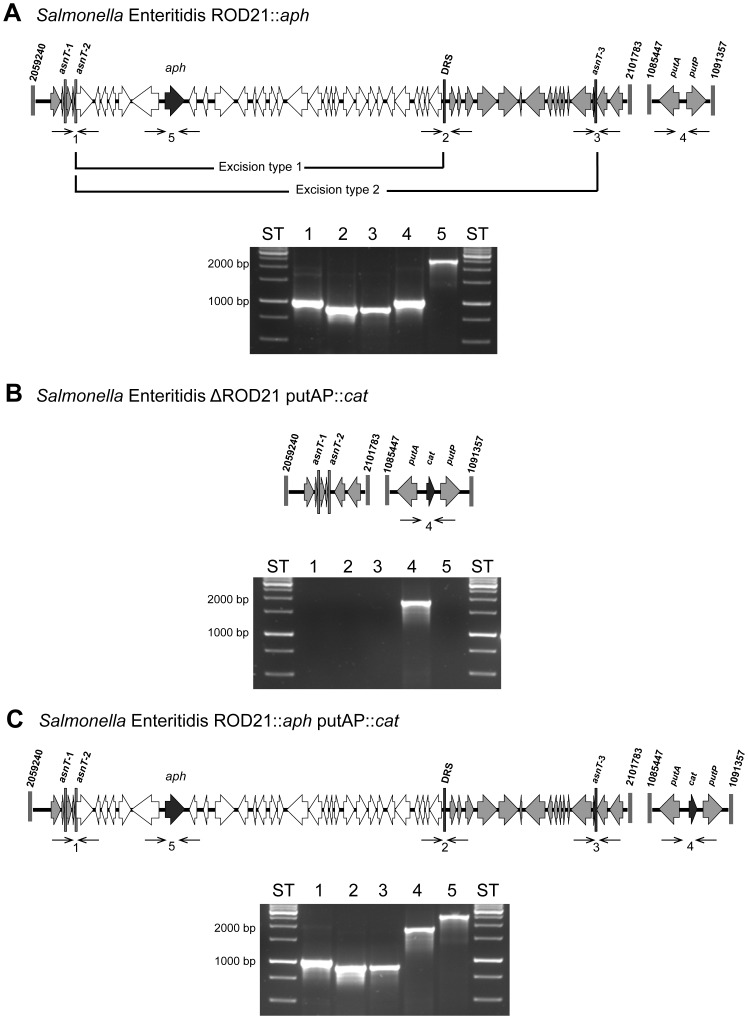
Schematic representation of the modified genetic regions of the strains used in the *S.* Enteritidis-to-*S.* Enteritidis mating assays. (**A**) Donor strain, showing excision type 1 and 2; (**B**) recipient strain and (**C**) transconjugant strain. Numbers above of each scheme represent the coordinates in the chromosome of *S.* Enteritidis. Asparagine tRNA genes are designated as *asnT-1*, *-2* and *-3*. DRS stands for Direct Repeated Sequence. Black arrows represent the inserted antibiotic gene (*aph* or *cat*). Thin arrows represent primers used for PCR verification of the strains: (1) left-end of ROD21 (1,011 bp), (2) DRS (914 bp), (3) right-end (903 bp), (4) insertion of *cat* gene (1,030 bp without *cat* insert, 1,943 bp with *cat* insert), and (5) insertion of *aph* gene (2,469 bp). Below each scheme, agarose gels show PCR products obtained for the recipient and transconjugant *S.* Enteritidis strain.

**Figure 2:**
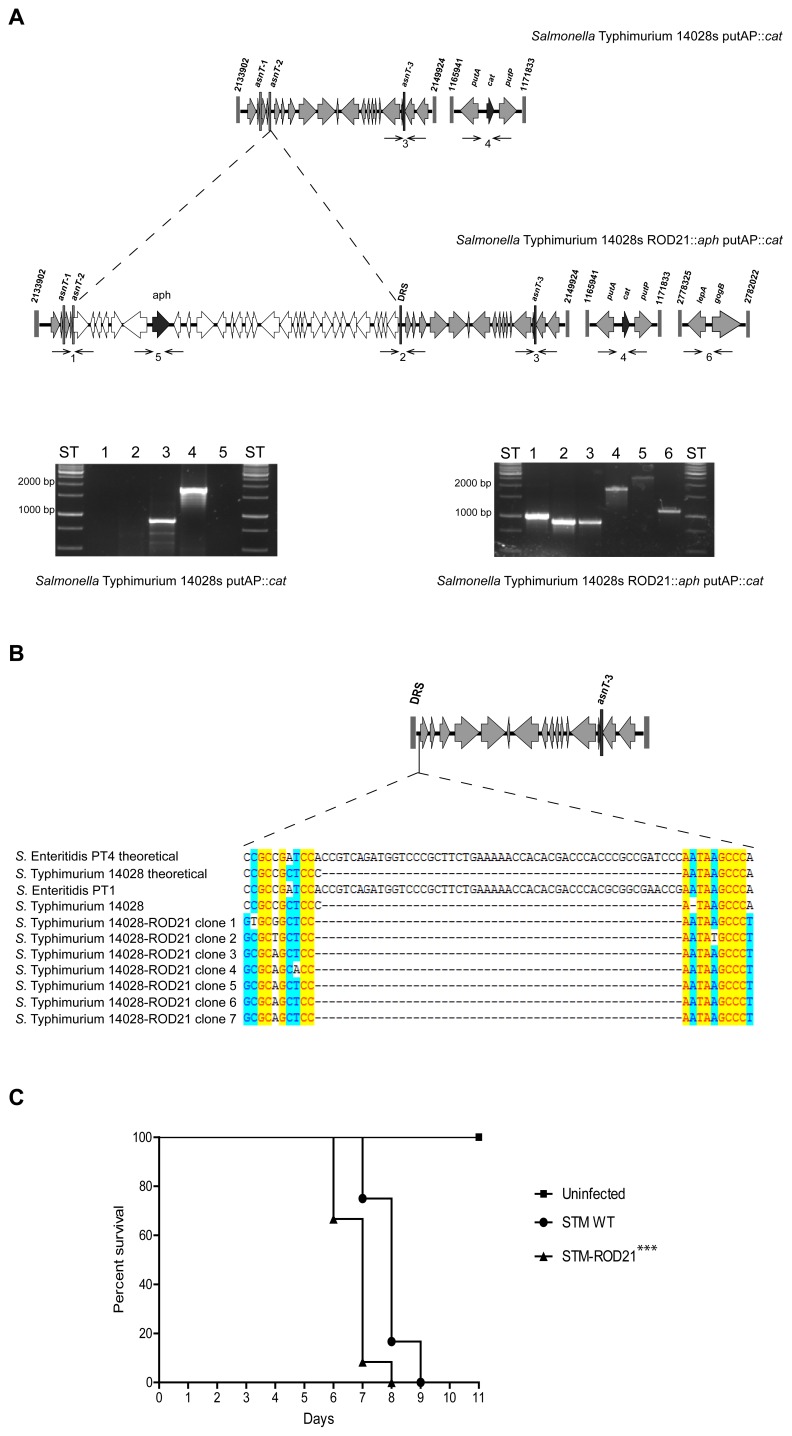
Transfer of ROD21 to *S.* Typhimurium. (**A**) Scheme of the genome of the *S.* Typhimurium recipient strain with *cat* gene inserted in the putAP region (black arrow) and double-resistant strain with *aph* gene inserted in ROD21 and *cat* gene inserted in the putAP region (black arrows). Numbers above each scheme represent the coordinates in the chromosome of *S.* Typhimurium. Asparagine tRNA genes are designated as *asnT-1*, *-2* and *-3*. DRS stands for Direct Repeated Sequence. Thin arrows represent primers used for PCR verification of the strains: (1) left-end of ROD21 (1,011 bp), (2) DRS (914 bp), (3) right-end of ROD21 (903 bp), (4) insertion of *cat* gene (1,838 bp), (5) insertion of *aph* gene (2,469 bp), and (6) left-end of Gifsy-1 (1,110 bp). Below the scheme, agarose gels show PCR products obtained for the recipient and transconjugant *S.* Typhimurium strains. (**B**) Schematic representation showing the location of a 50 bp region present in *S.* Enteritidis, downstream DRS, but absent in *S.* Typhimurium. The figure below shows the alignments of the theoretical sequence of the above mentioned region for *S.* Enteritidis PT4 and *S.* Enteritidis 14028 and the experimental sequences obtained for *S.* Enteritidis PT1, *S.* Typhimurium 14028 and 7 transconjugants of *S.* Typhimurium-ROD21 obtained in a representative transfer assay. (**C**) Survival of C57BL/6 mice intragastrically infected with 1×10^5^ CFU of *S.* Typhimurium wild type and *S.* Typhimurium-ROD21 strain. Data shown are the averages of three independent experiments, which included 4 mice per group. ***, P<0.05 Kaplan-Meier and Mantel-Cox post test.

The location of primers pairs from 1 to 6 is shown in Figures 1 and 2. Lowercase indicate the region that hybridizes to the 5′ or 3′ end of the pKD3 or pKD4 plasmids.

The strain *S.* Enteritidis ΔDRS ΔSEN1970 was generated by Lambda Red-mediated recombination, as described previously [9]. Using pKD4 plasmid as a template, a kanamycin resistance gene was amplified by PCR with the primers SEN 1970 (H1+P1) and asnT – SEN 1970 (H2+P2) to delete SEN1970 and asnT- SEN 1970 (H1+P1) with asnT – SEN 1970 (H2+P2) to delete DRS and SEN1970. The primers used to generate the PCR product from pKD4 were SEN 1980::Kan (H1+P1) with SEN 1980::Kan (H2+P2). Capital letters account for sequence that hybridizes with the *S.* Enteritidis chromosome and lowercase letters account for sequence that hybridizes with pKD4 plasmid. To generate strain *S.* Enteritidis ROD21::*aph*/pBAD-SEN1998, donor strain *S.* Enteritidis ROD21::*aph* was transformed by electroporation with either plasmid pBAD-empty or pBAD-SEN1998, as described previously [9] and seeded in solid LB medium supplemented with 50 µg/mL kanamycin and 100 µg/mL ampicillin.

### Molecular biology techniques

Bacterial genomic DNA was prepared using the phenol-chloroform method described by Sambrook *et al* [13]. Using genomic DNA from bacterial strains and primer pairs described in Table 2, PCR amplifications were performed in a MaxiGene Gradient Thermocycler (Axygen), using approximately 1 ng/mL of DNA, 1 nmol/mL of each primer, 0.2 mM deoxynucleoside triphosphates, 1.5 mM Magnesium Chloride and 50 U/mL of Taq DNA polymerase (Invitrogen), using standard PCR amplification cycles. PCR products were resolved by electrophoresis in 1% agarose gels containing 0.5 µg/mL ethidium bromide and visualized under a UV light transiluminator (UVP, Inc).

### Broth mating assays

To assess the potential for horizontal transfer of ROD21 in LB broth (1% tryptone, 0.5% yeast extract, 1% NaCl), mates were conducted as described previously by Coburn *et al* [14]. In these experiments, strain *S.* Enteritidis ROD21::*aph* (donor) and *S.* Enteritidis ΔROD21 putAP::*cat* (recipient) were grown to stationary phase in LB broth, washed with phosphate-buffered saline (PBS) pH 7.4 before mixing 0.15 mL of *S.* Enteritidis ROD21::*aph* and 0.45 mL of *S.* Enteritidis ΔROD21 putAP::*cat* with 4.5 mL of pre-warmed LB broth. After incubation at 37°C for 4 h, aliquots were removed for quantitative determination of donors, recipients and transconjugants by seeding on solid LB medium supplemented with the appropriate antibiotics. Donor *S.* Enteritidis ROD21::*aph* was selected on solid LB medium supplemented with kanamycin, recipient *S.* Enteritidis ΔROD21 putAP::*cat* was selected on solid LB medium supplemented with chloramphenicol and transconjugants were selected on solid LB medium supplemented with kanamycin and chloramphenicol, or kanamycin and trimethoprim. Transfer frequency measurements were calculated by dividing the number of transconjugants by the viable counts of donor cells in the mating mixture, determined at the end of the 4 h period. Using the same approach, we performed broth mating assays to determine whether ROD21 was able to transfer to the other recipient strains as mentioned above: *S.* Typhimurium 14028s putAP::*cat*, *S.* Typhimurium *phoN::dhfr*, H10407 putAP::*cat* and EDL933 putAP::*cat* and *E. coli* DH5α NalR.

To rule out transfer of ROD21 by naked DNA uptake due to lysis of donor cells, we purified DNA from the donor strain *S.* Enteritidis ROD21::*aph* using the phenol-chloroform methodology. Then, 10 ng, 100 ng and 1,000 ng were resuspended in LB medium and mixed with 450 µL of stationary phase, PBS-washed, *Salmonella* recipient strains. The mixture was inoculated in 4.5 mL pre-warmed LB medium in the same conditions described above. To test whether ROD21 was transferred by phage transduction, a liquid culture of the donor strain *S.* Enteritidis ROD21::*aph* was filtered using a 0.2 µm pore size polycarbonate filter (Orange Scientific Braine-l'Alleud, Belgium) and the supernatants containing phages were treated with 10 units of RQ1 DNase (Promega, Madison, WI). For these experiments, 150 µL of the treated supernatants were mixed with 450 µL of stationary phase, PBS-washed, *Salmonella* or *E. coli* recipient strains. Then, the mixture was inoculated in 4.5 mL pre-warmed LB medium under the same conditions described above.

### Filter mating assays

Filter mating assays were carried out employing a donor to recipient ratio equal to 1∶10. Stationary phase cultures of the donor (*S.* Enteritidis ROD21::*aph*, *S.* Typhimurium ROD21::*aph trg::mudQ* pSLT^+^ or *S.* Typhimurium ROD21::*aph trg::mudQ* pSLT^−^) and recipient (*S.* Enteritidis ΔROD21 putAP::*cat*, *S.* Typhimurium 14028s putAP::*cat*, *S.* Typhimurium *phoN::dhfr* pSLT^+^, *S.* Typhimurium *phoN::dhfr* pSLT^−^, *E. coli* H10407 putAP::*cat*, *E. coli* EDL933 putAP::*cat* or *E. coli* DH5α NalR) bacteria were mixed in LB medium (0.5 mL of the donor and 4.5 mL of the recipient) and then collected on a 0.2 µm pore size mixed cellulose/ester membrane filter (Advantec). The filter was placed with bacteria side down on a LB agar plate and incubated at 37°C for 4 h. Bacterial cells were harvested from the filter and resuspended to a final volume of 1 mL in LB and seeded on LB agar plates containing the respective antibiotics for selection. Transconjugants were selected with kanamycin and chloramphenicol or kanamycin and nalidixic acid. Transfer frequency measurements were obtained by dividing the number of transconjugants by the viable counts of donor cells in the mating mixture, determined at the end of the mating assay.

### Oxidative stress, temperature and pH treatments

To evaluate whether environmental conditions, such as oxidative stress, temperature and pH could influence the transfer of ROD21, we measured the transfer frequency in bacteria grown in the presence of hydrogen peroxide (H_2_O_2_), in bacteria grown at 23°C, 37°C or 43°C and in bacteria grown at pH 7 or pH 5. All treatments were performed in separate experiments. As mentioned above, donor and recipient bacteria were grown overnight at 37°C to stationary phase and aliquots were co-incubated for 4 h in LB medium. For oxidative stress assays, co-cultures were incubated at 37°C in LB medium supplemented with 0.25 mM H_2_O_2_ (Merck). For temperature treatments, co-cultures were incubated in LB medium at 23°C, 37°C or 43°C. Finally, for pH treatments, donor and recipient bacteria were grown at 37°C in LB medium adjusted to pH 5 with HCl (Merck). The frequency of ROD21 transfer was determined as mentioned above.

To detect ROD21 excision in the conditions described above, 1 mL of each bacterial culture was used to prepare genomic DNA and detection of ROD21 excision was performed with TaqMan MGB probes and TaqMan Universal PCR Master Mix (Applied BioSystems), as recommended by the manufacturer. Primers and probes used to detect ROD21 excision (*attB-1*) were: attB1-nested-RT-Fw, attB1-nested-RT-Rev and attB-1 probe (Table 2). Primers and the probe used to detect *rpoD* were: rpoD-RT-Fw, rpoD-RT-Rev and rpoD probe (Table 2). The reaction mixture contained 1 µL of genomic DNA as a template, 0.3 pmol/µL of each primer and 0.3 pmol/µL of TaqMan MGB probes. Standard curves for *attB-1* and *rpoD* were generated using serial dilutions of a plasmid containing the corresponding PCR fragment for *attB-1* and *rpoD*. Thermal cycling conditions were: 1 cycle of 5 min at 50°C and 10 min at 95°C and 40 cycles of 15 sec at 95°C, 15 sec at 58°C and 1 min at 60°C. The copy number of *attB-1* and *rpoD* was calculated as follows: the number of *attB-1* and *rpoD* copies was determined by the ratio between the amount of DNA in a sample and the weight of one molecule of the plasmid with the insert. To graph the standard curve, the value of the threshold cycle (Ct) was confronted with the log10 of the initial copy number of each sample, generating a linear relationship to calculate the number of copies in the sample, which has a specific Ct. The values were expressed as the ratio of *attB-1* copy number/*rpoD* copy number and for each condition evaluated, the fold increase over the basal condition (pH 7, 37°C and absence of hydrogen peroxide) was plotted.

### Survival assays

Groups of 4 male C57BL/6 mice (5–6 week age) were used to evaluate the virulence of WT *S.* Typhimurium and the strain *S.* Typhimurium-ROD21. To perform infection assays, bacteria were grown in LB medium at 37°C until an OD_600_ equal to 0.6 was reached. The volume of bacterial culture containing 1×10^5^ CFUs was centrifuged in a refrigerated microcentrifuge (CT15RE Hitachi) at 10,000× *g* for 5 minutes. Bacterial pellets were thoroughly resuspended in 200 µL of PBS and used to infect mice intragastrically. Serial dilutions of the bacterial inoculum were seeded in LB plates to corroborate infective doses. After infection, survival rate was recorded every 12 h. Mice that lost more than 25% of their initial body weight after infection were euthanized.

### Statistical analyses

For all experiments, 3 independent assays were performed (n = 3). Data and statistical analysis were performed using Prism 4 software (Graph Pad Software, Inc.). For statistical analyses unpaired Student's t test, Wilcoxon signed rank test and Mann-Whitney test were used when the data were not in a Gaussian distribution, or two ways ANOVA, when the distribution of data was Gaussian. The survival analyses were performed with Kaplan-Meier with Mantel-Cox post-test. In any case, p values below 0.05 were considered statistically significant.

## Results

### ROD21 is transferred between Salmonella strains

As we have shown previously, ROD21 can excise from the chromosome of *S.* Enteritidis in at least 2 different ways (excision type 1 and excision type 2) [6]. When excision type 1 takes place, genes from SEN1970 to SEN1999 excise from the chromosome and form an episomal element. On the other hand, excision type 2 promotes the excision of an episomal element including genes SEN1970 to SEN2011 (Figure 1A). To evaluate which episomal element can be transferred from *S.* Enteritidis to a ROD21-deficient *S.* Enteritidis strain, we generated donor and recipient strains. As shown in Figure 1A, to generate the donor strain, a kanamycin resistance gene (*aph*) was inserted between genes SEN1975 and SEN1976 in a strain of *S.* Enteritidis that has ROD21 inserted on the chromosome (*S.* Enteritidis ROD21::*aph*). Similarly, to generate a recipient strain, a chloramphenicol resistance gene (*cat*) was inserted between genes SEN0986A (*putA*) and SEN0987 (*putP*) in a *S.* Enteritidis strain that had lost ROD21 due to excision type 2 (*S.* Enteritidis ΔROD21 putAP::*cat*) (Figure 1B). To evaluate whether ROD21 was transferred from the donor to the recipient strain, we co-cultured both strains as described in Materials and Methods and selected transconjugants *S.* Enteritidis. In these assays, we found transconjugant bacteria, suggesting that ROD21 was effectively transferred from the donor to the recipient strain. To corroborate that these transconjugant strains of *S.* Enteritidis have incorporated ROD21 in their chromosomes, we detected the insertion of this pathogenicity island by PCR at the *attB* site in the chromosome of the recipient strain. As shown in Figure 1C, we observed that the recipient strains had incorporated ROD21 at the *attB* site. Interestingly, all transconjugant bacteria evaluated in these assays acquired genes SEN1970 to SEN2011, suggesting that the episomal element was generated due to excision type 2, transferred from the donor strain and successfully integrated in the chromosome of recipient bacteria.

### ROD21 is transferred from S. Enteritidis to S. Typhimurium

Next, we evaluated whether ROD21 can be transferred from *S.* Enteritidis to *S.* Typhimurium. Recipient strains of *S.* Typhimurium were generated by insertion of the *cat* gene between the genes STM14_1278 (*putA*) and STM14_1281 (*putP*), respectively (Figure 2A). Recipient and donor strains were mixed and incubated in LB medium, as described above. In these assays, we obtained recipient *S.* Typhimurium strains that acquired ROD21 from donor *S.* Enteritidis. To corroborate that these transconjugant *S.* Typhimurium strains effectively acquired ROD21, the borders of the island and several regions inside ROD21 were amplified by PCR (Figure 2A). To demonstrate that the double resistant *S.* Typhimurium strains had acquired ROD21 and to rule out the transfer of the chloramphenicol resistance gene from *S.* Typhimuriun to *S.* Enteritidis, we successfully amplified segments of DNA by PCR that were present only in *S.* Typhimurium but absent in *S.* Enteritidis (genes in the prophage Gifsy-1). These PCR reactions were performed in all the transconjugant colonies obtained in each assay and in all of them the prophage Gifsy-1 was detected (data not shown). These data support the notion that ROD21 can be transferred from *S.* Enteritidis to *S.* Typhimurium.

To determine which episomal ROD21 element was transferred and integrated in the chromosome of *S.* Typhimurium (episomal ROD21 produced either by excision type 1 or type 2), we amplified a portion of the DNA included between *attR* and the *asnT-2* of ROD21, which is present in the episomal element generated by excision type 2 (Figure 2B) and shared by *S.* Enteritidis and *S.* Typhimurium. In that region there is a fragment of 50 bp that is present only in *S.* Enteritidis but absent in *S.* Typhimurium and therefore allowed us to discriminate whether or not the DNA fragment between DRS and *asnT-2* present in the genome of the transconjugant *S.* Typhimurium came from *S.* Enteritidis. Thus, we sequenced the PCR product generated to detect the 50 bp that are present in *S.* Enteritidis but absent in *S.* Typhimurium. All the *S.* Typhimurium transconjugants obtained in these assays were sequenced and none of them showed the sequence specific for *S.* Enteritidis (Figure 2B). These data suggest that the acquired episomal element from *S.* Enteritidis was the one generated due to excision type 1 and that the integration site of ROD21 in *S.* Typhimurium was *asnT-2*.

To determine if the virulence of *S.* Typhimurium could be affected due to the acquisition of ROD21, we infected C57BL/6 mice intragastrically with both *S.* Typhimurium and *S.* Typhimurium-ROD21 and measured survival. As shown in Figure 2C, we observed that mice infected with *S.* Typhimurium-ROD21 showed reduced survival as compared to mice infected with WT *S.* Typhimurium. These results support the notion that acquisition of ROD21 by *S.* Typhimurium increase bacterial virulence, indicating that the genes contained in this pathogenicity island can work as virulence factors.

### Excision of ROD21 is required for the transfer to recipient strains

**Figure 3:**
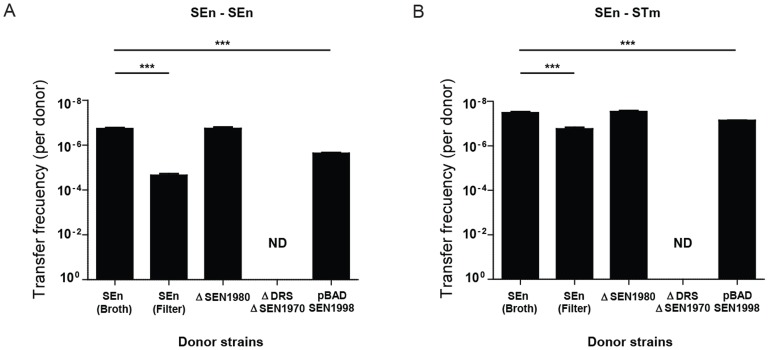
ROD21 transfer requires excision. Transfer frequency of ROD21 from donor *S.* Enteritidis, from *S.* Enteritidis ΔDRSΔSEN1970, from *S.* Enteritidis ΔSEN1980 and from *S.* Enteritidis/pBAD-SEN1998 supplemented with arabinose to (**A**) recipient *S.* Enteritidis and (**B**) recipient *S.* Typhimurium. Graph shows the frequency of ROD21 transfer, expressed as the ratio between transconjugant bacteria and the total amount of donors calculated in each assay. Results are the average of four independent experiments (Y axis are expressed as reverse of log values). ***, P<0.001, Student's *t* test. ND = Non-detected.

To determine if the transfer of ROD21 requires the excision of this pathogenicity island and to rule out chromosomal transfer, we used a *S.* Enteritidis strain unable to excise ROD21 that we have described in a previous study [9]. This bacterial strain lacks the ORF SEN1970, which encodes for an integrase belonging to the family of P4 coliphages. Further, this strain lacks the DRS/*attL* sequence required for excision. When transfer assays were performed using this mutant strain, no transconjugant bacteria were obtained (Figure 3A). In addition, we tested ROD21 transfer using a *S.* Enteritidis strain that excises ROD21 with higher frequency due to over expression of SEN1998, an ORF that encodes for a putative excisionase. We have previously shown that a strain of *S.* Enteritidis harboring the plasmid pBAD-SEN1998 showed enhanced ROD21 excision due to arabinose-induced SEN1998 overexpression (Figure S1) [9]. As shown in Figure 3, the transfer rate of ROD21 from *S.* Enteritidis/pBAD-SEN1998 to recipient strains increased by almost 13 times (Figure 3A–B), suggesting that ROD21 excision is required for its transfer.

### ROD21 is transferred from donor to recipient strains by conjugation

After showing that ROD21 could be transferred from *S.* Enteritidis to *S.* Enteritidis and *S.* Typhimurium, the HGT mechanism responsible for mobilization of this pathogenicity island was explored. Recipient *S.* Enteritidis was incubated with 10 ng, 100 ng or 1,000 ng of purified DNA from donor strain and with the filtered supernatant of the donor strain, as described in Materials and Methods. In both cases we failed to obtain bacteria resistant to both antibiotics (data not shown), ruling out that ROD21 could be transferred by natural transformation or by phage transduction. However, when we performed filter mating assays, as described in Materials and Methods, we found a higher frequency of transfer from *S.* Enteritidis to both *S.* Enteritidis and *S.* Typhimurium (Figure 3A–B). These data suggest that ROD21 transfer takes place by means of bacterial conjugation.

To evaluate if ROD21 encodes its own conjugation system, we generated a *S.* Enteritidis strain lacking ORF SEN1980, which encodes a protein similar to *mobA* from the *E. coli* plasmid RSF1010 and which has been involved in mobilization of genetic elements. We observed that deletion of SEN1980 did not alter the transfer frequency of ROD21 (Figure 3A). This result suggests that ROD21 transfer relies in a conjugation system encoded by genes outside this pathogenicity island. Supporting this hypothesis is the fact that *S.* Enteritidis harbors a virulence plasmid similar to the virulence plasmid pSLT of *S.* Typhimurium, which contains ORFs homologous to *tra* genes with an organization similar to the F plasmid [15]. Given that strains of *S.* Enteritidis lacking the virulence plasmid are not available, we tested whether absence of the virulence plasmid could affect the transfer of ROD21 in a *S.* Typhimurium strain lacking this plasmid [16], [17]. First, we tested *S.* Typhimurium ROD21::*aph trg::mudQ* pSLT^+^ as a donor strain and *S.* Typhimurium *phoN::dhfr* pSLT^+^ as recipient strain, and we effectively observed transfer of ROD21 between these bacteria, at a frequency equal to 1.1×10^−8^. Then, we used the strain *S.* Typhimurium ROD21::*aph trg::mudQ* pSLT^−^ as a donor strain and *S.* Typhimurium *phoN::dhfr* pSLT^−^ as a recipient strain. When co-culture assays in LB medium were performed, no transconjugant bacteria were obtained, suggesting that ROD21 transfer requires conjugative machinery, which is possibly encoded by the virulence plasmid.

### Changes in temperature and pH affect the frequency of ROD21 transfer

**Figure 4:**
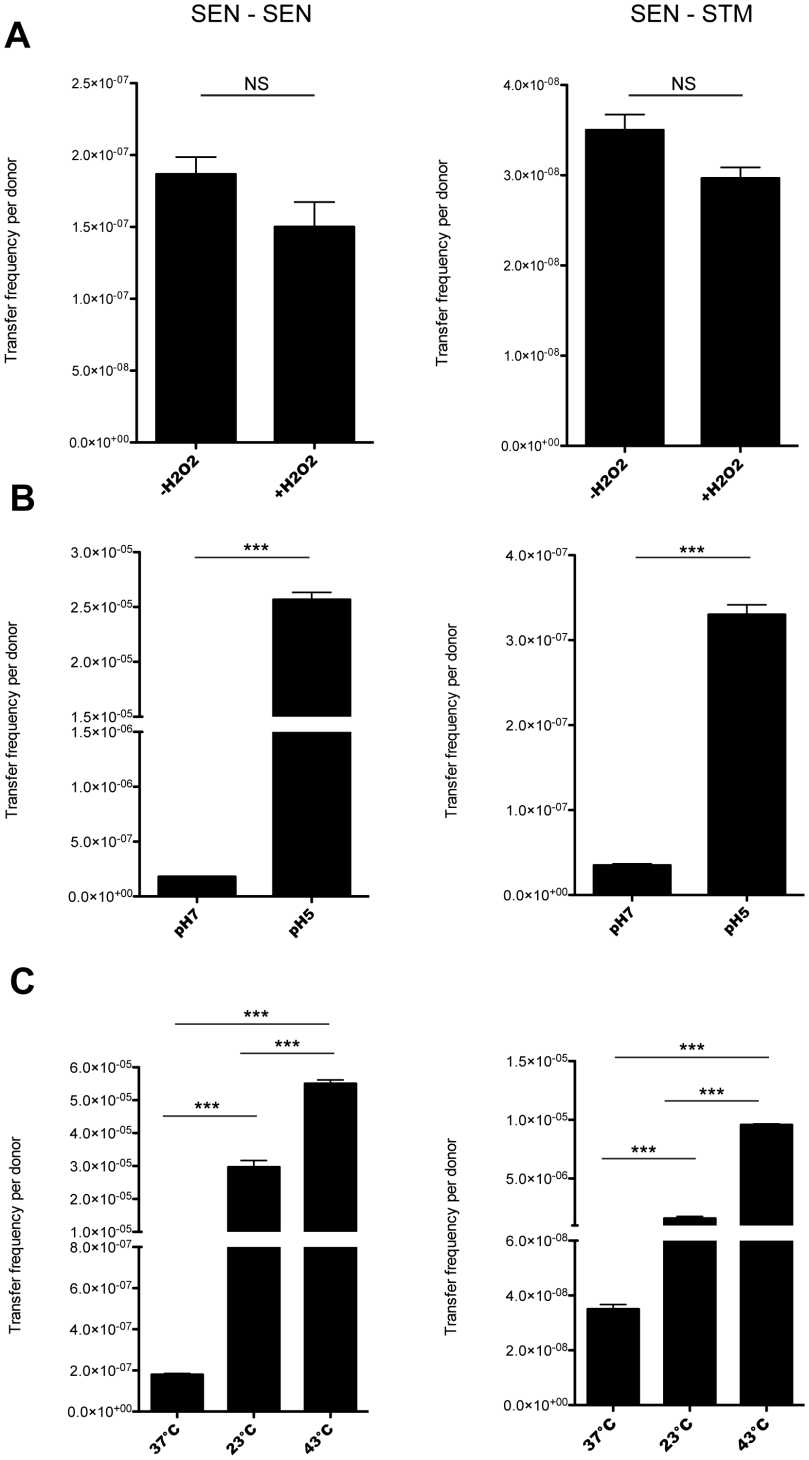
Transfer of ROD21 to *S.* Enteritidis and *S.* Typhimurium in different environmental conditions. Strains were subjected to transfer assays in LB medium and (**A**) LB medium supplemented with hydrogen peroxide, (**B**) pH 7 and pH 5, and (**C**) 23°C and 43°C. Error bars represent standard deviation from the mean (N = 3). NS = non-significant, Student t-Test (Two tailed). ***, p<0.001 one-way ANOVA and Tukey post-test.

Next, we evaluated what environmental factors could influence the transfer frequency of ROD21. Previously we had reported that ROD21 excision increased when bacteria were exposed to hydrogen peroxide and when bacteria resided inside phagocytic cells [6]. Therefore, we performed transfer assays in the presence of H_2_O_2_. As shown in Figure 4A, no significant changes in the transfer frequency of ROD21 were observed neither to *S.* Enteritidis nor to *S.* Typhimurium, suggesting that in the conditions used in this study oxidative stress failed to alter the transfer frequency of this pathogenicity island. Next, we evaluated whether changes in temperature or pH could affect the transfer of ROD21. Transfer assays were performed incubating the recipient and donor strains at either pH 7 or 5 and observed a significant increase of the transfer frequency from *S.* Enteritidis to both *S.* Enteritidis (Figure 4B, left panel) and *S.* Typhimurium (Figure 4B, right panel). Further, the frequency of ROD21 transfer was measured at 37°C, 23°C and 42°C, and observed that the transfer of this pathogenicity island from *S.* Enteritidis to *S.* Enteritidis (Figure 4C, left panel) or to *S.* Typhimurium (Figure 4C, right panel) increased when bacteria were incubated at 23°C and 43°C, as compared to 37°C.

**Figure 5:**
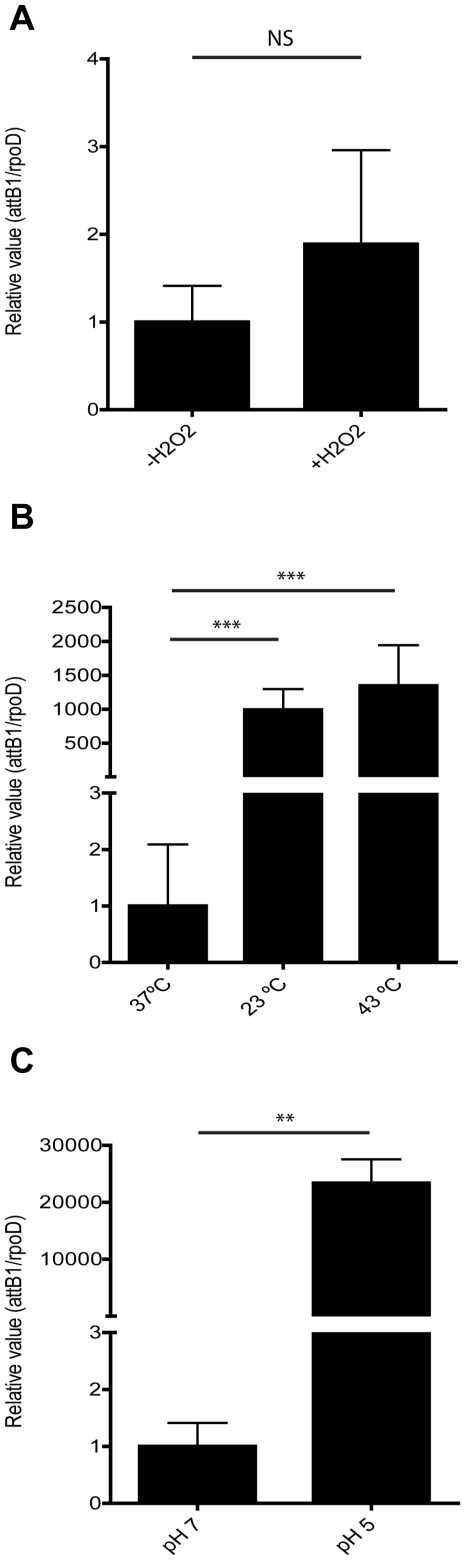
Excision of ROD21 in different conditions. Donor *S.* Enteritidis strain was grown in LB medium and (**A**) LB medium supplemented with hydrogen peroxide, (**B**) lower pH (pH 5), and (**C**) lower and higher temperatures (23°C and 43°C respectively) in order to evaluate excision of ROD21. The frequency of *attB1* excision was quantified by qPCR using genomic DNA and was expressed as a relative value equal to the ratio between the copy number of *attB1* over the copy number of *rpoD* gene. Error bars represent standard deviation from the mean (N = 3). ***, P<0.001, **, P = 0.016 (one-way ANOVA and Tukey's post-test).

To evaluate whether the increased transfer of ROD21 correlated with an enhanced ROD21 excision, we used qPCR to quantify the amount of *attB* sequence in bacteria grown in the presence of H_2_O_2_, as well as in bacteria grown at different temperatures and at different pH values. As shown in Figure 5, the presence of H_2_O_2_ (A) produced a non-significant increase of ROD21 excision. However, when bacteria were exposed to 23°C, 43°C (B) or pH 5 (C) the level of ROD21 excision showed a significant increase. As expected, higher excision was detected when we observed increased transfer. These results suggest that changes in environmental conditions could increase the excision and transfer frequency of ROD21.

### ROD21 is not transferred to E. coli

Because the organization and sequence of the genes coding for asparagine tRNA in the genome of *E. coli* is similar to *S.* Enteritidis (Figure S2), we thought that ROD21 could be transferred to *E. coli* and integrated in its chromosome. To evaluate this possibility, we performed transfer assays using *S.* Enteritidis as a donor and *E. coli* as a recipient strain (Figure S2). However, no *E. coli* strains that were resistant to both antibiotics were obtained after co-cultures between donor *S.* Enteritidis and each of the recipient strains of *E. coli*. Moreover, the nalidixic acid resistant *E. coli* DH5α was used as an avirulent and permissive strain as a recipient, but failed to produce transconjugants. We also tested ROD21 transfer from *S.* Enteritidis to each of the donor *E. coli* strains at different pH and temperatures and did not detect double-resistant strains (data not shown). These data suggest that *E. coli* is not permissive for ROD21 transfer from *S.* Enteritidis.

## Discussion

HGT has been highlighted as one of the main mechanisms responsible for bacterial microevolution defined as a rapid adaptation to environmental changes [18]. Along these lines, *Salmonella enterica* is an important example because recent genome sequencing studies have suggested that about 10% of their genetic material has been acquired by HGT [4], [19]. These HGT elements have caused great impact on the variability of the *Salmonella* genus, in which more than 2,500 serovars have been identified.

The aim of this study was to evaluate the transfer of the pathogenicity island ROD21, present in the chromosome of *S.* Enteritidis, to other strains of *Salmonella*. ROD21 was described as a region present in *Salmonella* serovars Gallinarum, Dublin, Typhi and Enteritidis [5]. These data suggested that these serovars had acquired ROD21 by HGT. We have reported that ROD21 is an unstable element, due to its ability to undergo spontaneous excision from the bacterial chromosome during growth in liquid culture medium and during infection of murine immune cells [6]. It has been reported that pathogenicity islands, that are capable of spontaneous excision from the genome of Gram positive and Gram negative bacteria, have the ability to be transferred to other bacteria [14], [20], [21].

By using transfer assays in liquid LB medium, we obtained recipient strains of *S.* Enteritidis that have acquired the pathogenicity island, which integrated to the chromosome of these bacteria. *S.* Typhimurium was also found to acquire and integrate the pathogenicity island into its genome. Furthermore, our results suggest that transfer of ROD21 requires excision of the pathogenicity island from the chromosome. Consistently, conditions that increase ROD21 excision also cause an increase of the transfer frequency of this genetic element. These results support the notion that ROD21 is an unstable and mobile genetic element that can be transferred between *Salmonella* strains.

According to our previous studies, it is likely that ROD21 excises from the *S.* Enteritidis chromosome by at least two different recombination events [6], leading to the generation of two episomal elements. Here, the strain of *S.* Enteritidis used as a recipient strain spontaneously lost the island due to excision type 2 (Figure 1). We found that transconjugant *S.* Enteritidis recovered the entire region (comprising genes from SEN1970 to SEN2011). Previously, we reported that ROD21 loss was detectable only in bacteria that underwent excision type 2 [6], suggesting that the episomal element produced by this event of recombination was transferred and integrated with higher frequency than episomal elements generated by recombination between the different *asnT* genes or between *asnT* genes and the DRS. While these different recombination events could generate at least 5 different episomal elements, we found that the episomal element generated by excision type 2 was able to be integrated in the chromosome of *S.* Enteritidis and detected using our experimental approach.

In the case of *S.* Typhimurium, this serovar could act as recipient for ROD21 because the organization of the *asnT* genes was similar to *S.* Enteritidis, but only lacks this genetic island (genes SEN1970 to SEN1999) [6]. Analysis of double resistant transconjugant bacteria showed that they had acquired the complete pathogenicity island. Moreover, sequence assays suggest that transconjugant *S.* Typhimurium incorporated ROD21 by site-specific recombination of the episomal element formed by excision type 1.

When experiments were performed using *E. coli* as recipient bacteria, no transconjugant bacteria were obtained. It was previously described that one of the greatest barriers to the transfer of genetic material is the presence of restriction systems, especially for the acquisition of DNA from a different species [22], [23]. However, we did not obtain transconjugant bacteria when we used the *E. coli* strain DH5α, which has a mutation in the Endonuclease I gene (*endA*). It has also been proposed that the main obstacle to the acquisition of genetic material between *E. coli* and *Salmonella* is the level of sequence identity between the donor and recipient bacteria, where the SOS system would play an important role [24], [25], [26]. These explanations could account for the lack of ROD21 integration in the genome of recipient *E. coli* strains.

As mentioned above, transfer of genetic material may occur by transformation, transduction or conjugation. The presence in ROD21 of CDS with homology to proteins involved in bacterial conjugation, such as MobA, TraD, PilS and PilV, suggests conjugation as the mechanism responsible for the transfer. However, it is known that complete operons encoding the conjugation machinery are required to mediate the transfer of unstable episomal elements [27]. Consistent with this notion, deletion of the *mobA* homolog SEN1980 did not alter the frequency of ROD21 transfer in our assays. By analyzing the mechanism by which ROD21 is transferred, we ruled out natural transformation and transduction. Results of filter transfer assays, which increase the frequency of transfer to *S.* Enteritidis and *S.* Typhimurium, in two and one order of magnitude respectively, suggest that the event of mobilization occurs via conjugation. This increase is possibly due to a higher probability of intimate bacterial contact.

To date, many mobile genetic elements have been classified as Integrative and Conjugative Elements (ICE) because they possess the complete machinery to excise and transfer by themselves to other bacteria [28], [29], [30]. When we used a strain of *S.* Typhimurium lacking the virulence plasmid pSLT, which has been described as similar to F plasmid [31], transfer of ROD21 did not occur. This result supports the notion that ROD21 requires conjugative machinery, likely provided *in trans*, to transfer to other bacteria. Some genetic elements capable of being transferred have been classified as Integrative and Mobile Elements (IME), despite lacking the genes encoding for the transfer machinery in their sequence. Such is the case of *Salmonella* genomic island 1 [32], thus it would seem appropriate to classify ROD21 as an IME.

As we have previously shown, environmental conditions can affect ROD21 excision [6], suggesting that they might modulate the transfer of this pathogenicity island. When analyzing the effect of the temperature, we observed an increase in the efficiency of ROD21 transfer from *S.* Enteritidis to *S.* Typhimurium both at 23°C and 43°C. Consistent with our data, transfer of plasmid R27 from *E. coli* increases at low temperatures (25°C to 30°C) due to an H-NS-mediated overexpression of genes involved in mobility [33]. CDS SEN1993 encoded in ROD21 shows significant sequence homology with the H-NS protein, which could explain the increased transfer frequency in response to temperature changes. Although it has been described that high temperatures decrease the transfer of genetic elements, the natural reservoir of *S.* Enteritidis is poultry, whose body temperature is approximately 43°C [34]. Thus, it is possible that transfer of ROD21 in poultry is a constant and dynamic phenomenon, possibly to ensure the presence of the pathogenicity island in the chromosome of *S.* Enteritidis.

It is known that pH can affect the transfer of genetic elements from one bacterium to another [35], [36], [37]. *Salmonella* passes through the digestive tract encountering various pH values, which could be an important aspect for the infectious cycle. Furthermore, pH modulation of ROD21 transfer could play a role while located in phagosomes inside phagocytic cells, which usually show low pH. Consistent with this notion is the observation that the transfer of ROD21 to *S.* Enteritidis and *S.* Typhimurium peaked at pH 5. It is likely that this process is modulated by the differential expression of genes involved in genetic transfer at various pH values. In agreement with these observations, we detected higher levels of ROD21 excision in the conditions where we observed higher transfer.

In conclusion, our data suggest that ROD21 is an unstable pathogenicity island that can be transferred to strains of *S.* Enteritidis and *S.* Typhimurium by conjugation. Such a transfer is a species-specific event that is affected by environmental conditions influencing both the natural life cycle of bacteria, as well as their infective process, ensuring the maintenance of this pathogenicity island by the bacterial population.

## Supporting Information

Figure S1 **Excision of ROD21 in donor strain overexpressing ORF SEN1998.** (A) Expression of excisionase (SEN1998) in wild type strain and donor strain transformed with the plasmids pBAD-empty or pBAD-SEN1998, when arabinose (1 mM) is added. The results are expressed as the ratio of SEN1998/*rpoD* expression (relative quantification or RQ). **, p<0.005 one-way ANOVA and Tukey post-test. (B) Quantification of excision of ROD21 in *S.* Enteritidis wild type, *S.* Enteritidis transformed with the plasmid pBAD-TOPO or pBAD-SEN1998, with or without arabinose (1 mM). The results are expressed as the ratio of *attB1*/*rpoD* (relative quantification or RQ). ***, p<0.001 one-way ANOVA and Tukey post-test.
(TIF)

Figure S2 **Generation of recipient** ***E. coli*** **strains.** (**A**) Scheme of the region of possible integration of ROD21 in *E. coli* strains H10407 and EDL933. Numbers on top of each scheme represent the coordinates in the chromosome of both strains. DRS stands for Direct Repeated Sequence (*attR*). Thin arrows represent primers used for PCR verification of the strains: (1) left-end of ROD21 (1,011 bp), (2) DRS (914 bp), (3) right-end of ROD21 (903 bp), (4) insertion of *cat* gene (1,958 bp) and (5) insertion of *aph* gene (2,469 bp). (**B**) Comparison of the sequences of asparagine tRNA genes of *S.* Enteritidis and *E. coli* strains used in this study.
(TIF)
