# Supplementary figures and images for: Conjugal Transfer of the Pathogenicity Island ROD21 in Salmonella enterica serovar Enteritidis Depends on Environmental Conditions
Source: PLoS One. 2014 Apr 4;9(4):e90626. doi: 10.1371/journal.pone.0090626 (PMC3976249; doi:10.1371/journal.pone.0090626)

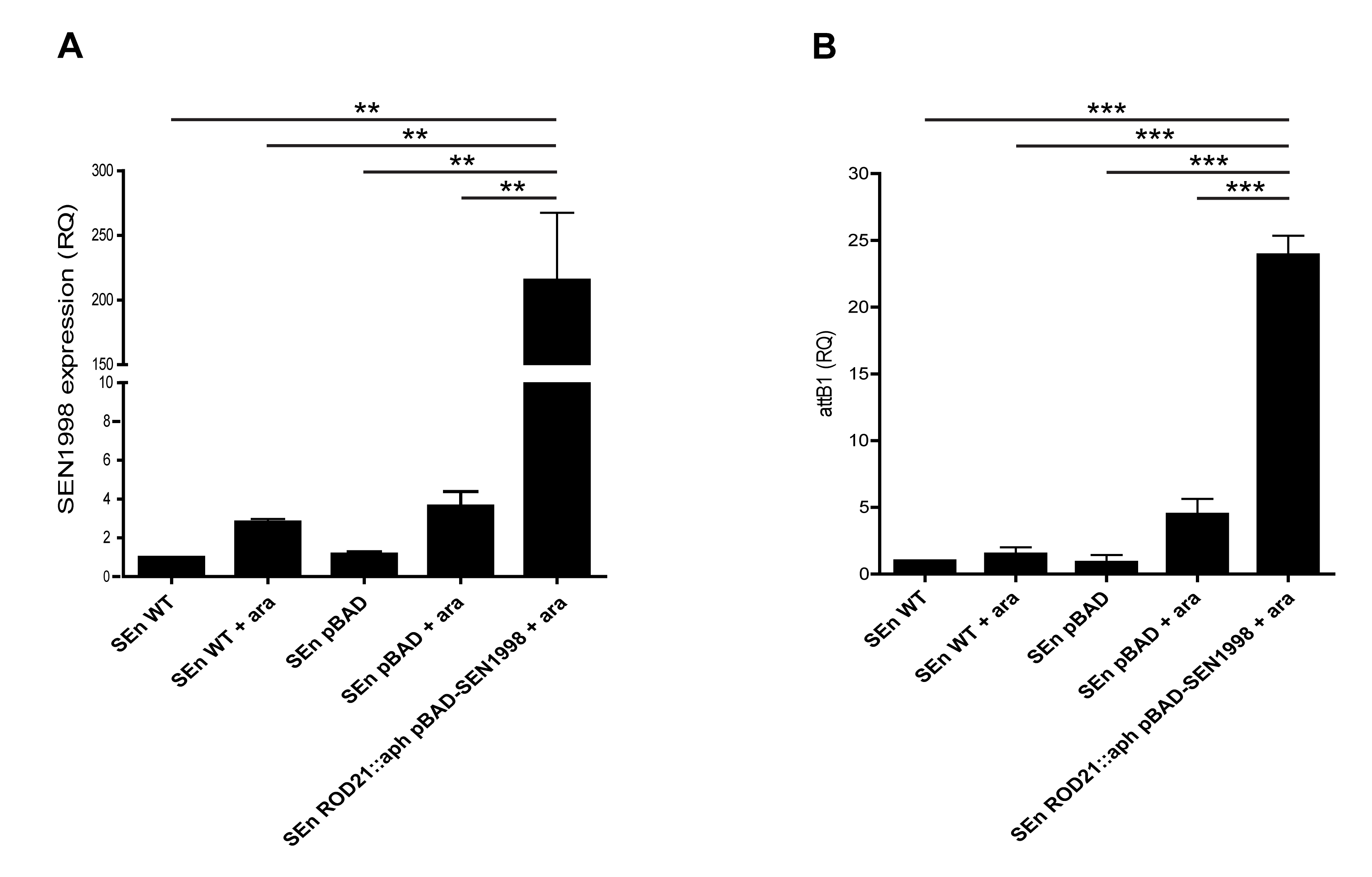

Supplement: Figure S1 — Excision of ROD21 in donor strain overexpressing ORF SEN1998. (A) Expression of excisionase (SEN1998) in wild type strain and donor strain transformed with the plasmids pBAD-empty or pBAD-SEN1998, when arabinose (1 mM) is added. The results are expressed as the ratio of SEN1998/rpoD expression (relative quantification or RQ). **, p<0.005 one-way ANOVA and Tukey post-test. (B) Quantification of excision of ROD21 in S. Enteritidis wild type, S. Enteritidis transformed with the plasmid pBAD-TOPO or pBAD-SEN1998, with or without arabinose (1 mM). The results are expressed as the ratio of attB1/rpoD (relative quantification or RQ). ***, p<0.001 one-way ANOVA and Tukey post-test. (TIF) [file pone.0090626.s001.tif]

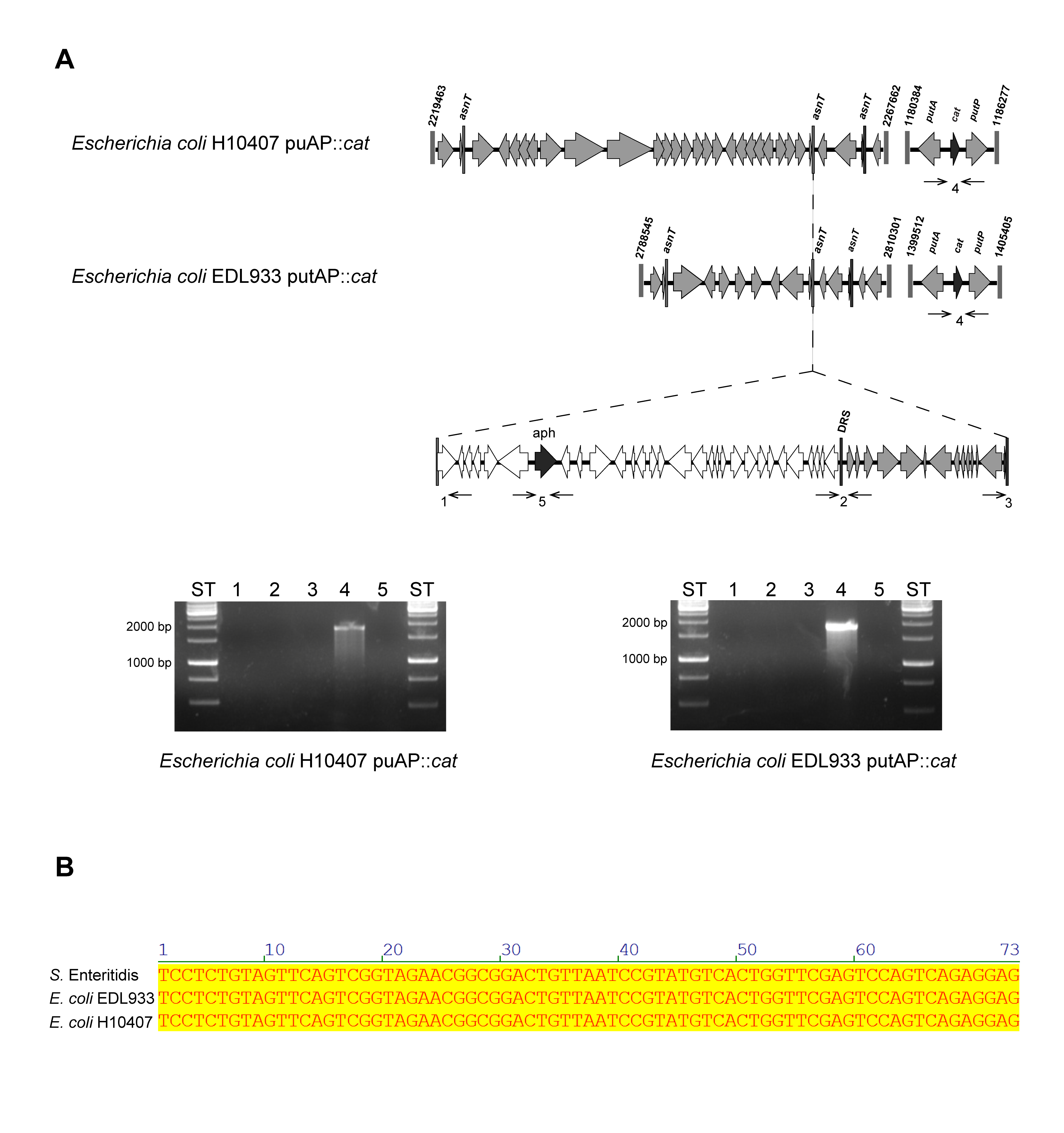

Supplement: Figure S2 — Generation of recipient E. coli strains. (A) Scheme of the region of possible integration of ROD21 in E. coli strains H10407 and EDL933. Numbers on top of each scheme represent the coordinates in the chromosome of both strains. DRS stands for Direct Repeated Sequence (attR). Thin arrows represent primers used for PCR verification of the strains: (1) left-end of ROD21 (1,011 bp), (2) DRS (914 bp), (3) right-end of ROD21 (903 bp), (4) insertion of cat gene (1,958 bp) and (5) insertion of aph gene (2,469 bp). (B) Comparison of the sequences of asparagine tRNA genes of S. Enteritidis and E. coli strains used in this study. (TIF) [file pone.0090626.s002.tif]
